# Enhanced Radiation Protection Devices in Every Fluoroscopic Laboratory: A Call to Action

**DOI:** 10.1016/j.jscai.2026.105398

**Published:** 2026-07-14

**Authors:** Farouc A. Jaffer

**Affiliations:** Cardiology Division, Massachusetts General Hospital, Mass General Brigham and Harvard Medical School, Boston, Massachusetts

**Keywords:** exposure, fluoroscopy, interventional cardiology, occupational health, radiation protection

For decades, physicians, nurses, technologists, and trainees have provided care for patients undergoing diagnostic and interventional fluoroscopic procedures. Such procedures span a spectrum of purpose and specialty care, including cardiovascular disease, radiology, gastroenterology, urology, gynecology, pain medicine, and orthopedic surgery. Importantly, providing care for such patients requires practitioners to protect themselves against the harmful effects of ionizing radiation. Radiation exposure increases the risks of malignancy, cataracts, bone marrow suppression, skin damage, organ atrophy, and cardiovascular disease.^1^^,^^2^ As a consequence, the United States and other countries have established that the standard-of-care for radiation exposure must be “as low as reasonably achievable” (“ALARA”).

For interventional cardiologists and other members of the cardiac catheterization laboratory, approaches to achieve ALARA radiation exposure include (1) operator and room shielding; (2) minimizing time of invasive procedures, fluoroscopy, and cineangiography; and (3) optimizing x-ray systems use to reduce exposure (ie, collimation, minimizing the distance between the x-ray detector and the patient, avoiding steep angulation, use of demagnified views, reducing acquisition frame rates, x-ray system dose optimization, employing radiation drapes, and use of real-time dosimeters to modify behavior).^2^ Despite these risk-mitigating strategies, interventional cardiologists remain among the most exposed medical professionals involved in fluoroscopic procedures.

The primary radiation shielding approach for interventional cardiologists involves the donning of personal lead garments (typically including an apron, vest, thyroid collar, and lead glasses), which effectively reduce radiation exposure to the covered areas; however, the weight of typical 0.5-mm lead garments imposes substantial physical strain. In addition, these garments do not provide complete protection, leaving the head, extremities, and portions of the neck exposed, particularly on the left side.^3^ In fact, a recent survey of interventional cardiologists reported that 66% had experienced musculoskeletal pain, 60% had at least 1 orthopedic injury, 6% had contracted cancer, and 5% had cataracts or skin injury.^4^ In addition, 17% of respondents reduced their time in the catheterization laboratory to reduce radiation exposure, and 20% indicated that if such harms were preventable, they would extend their occupational longevity by 10 years.^4^ Concerns also persist regarding incomplete fetal shielding. This combined set of factors negatively impacts operator longevity, physical and emotional health, and workforce recruitment—particularly among women.^5^

To address these challenges, a range of enhanced radiation protection devices (ERPDs) has been developed over the past several years, allowing additional reductions in radiation exposure by >90% (particularly when combined with real-time dosimetry). ERPDs offer the potential to eliminate or reduce the weight of personal lead garments; however, their adoption remains low, impeded by economic constraints, regulatory barriers, and insufficient advocacy. The time has come to change.

In this issue of *JSCAI*, Rizik et al^6^ presents a multisociety expert consensus statement that provides a moral, ethical, legal, and practical foundation to change the standard-of-care and to mandate the utilization of ERPDs in every fluoroscopic laboratory. This informative, compelling, and authoritative document depicts the hazards of radiation, current standard practice radiation protection approaches, and the respective health impact on practitioners. After detailing the specifications, function, and efficacy of many currently available ERPDs, the authors conclude that incorporating ERPD technology into every cardiac catheterization laboratory is the best option to achieve the federally mandated requirement for ALARA radiation exposure.

Considering this irrefutable perspective, can health care institutions afford not to mandate enhanced radiation protection in every fluoroscopic laboratory? In addition, given the established health hazards of radiation exposure, can procedurally based clinicians afford not to use ERPDs? The answer to both questions is a resounding “no!” Fortunately, the roadmap to implementation is clear, and all stakeholders (practitioners, health care leadership, professional societies, radiation safety regulators, and x-ray suite and ERPD manufacturers) have an opportunity and ethical mandate to revolutionize practice and to optimize safety by requiring the installation of ERPDs in every fluoroscopy suite. Given the impending shortage of interventional cardiologists^7^ and the expanding population of patients who will require invasive cardiovascular procedures,^8^ there is one clear road ahead: ERPDs in every fluoroscopy suite.

## Declaration of competing interest

Farouc A. Jaffer reports sponsored research: Canon, Siemens, Shockwave Medical, Teleflex, Mercator, Boston Scientific, HeartFlow, Neovasc, and Orbus Neich; consultant/speaker fees: Boston Scientific, Siemens, Magenta Medical, Philips, Biotronik, Mercator, Terumo, Abiomed, Shockwave Medical, DurVena, Intravascular Imaging Inc (i3), Medtronic, and FastWave; equity interest: Intravascular Imaging Inc (i3) and FastWave; and Massachusetts General Hospital licensing arrangements with Terumo, Canon, and SpectraWAVE, for which Farouc A. Jaffer has the right to receive royalties.
